# First record of *Triatoma vitticeps* (Hemiptera, Reduviidae) colonising a brick house in an urban area of a devastated Atlantic Forest fragment in Rio de Janeiro State, Brazil

**DOI:** 10.1590/0074-02760260038

**Published:** 2026-08-31

**Authors:** Jane Costa, Gabriela Ferreira Campos Guerra, Fernanda Cristina de Oliveira Firmino, Edmardo de Oliveira Campbell Junior, Cláudia Jucá da Silva, João Paulo Sales Oliveira-Correia, Dayse S Rocha, Cleber Galvão

**Affiliations:** 1 Fundação Oswaldo Cruz-Fiocruz, Instituto Oswaldo Cruz, Laboratório Nacional e Internacional de Referência em Taxonomia de Triatomíneos, Rio de Janeiro, RJ, Brasil; 2 Secretaria Municipal de Meio Ambiente, São José do Vale do Rio Preto, RJ, Brasil; 3 Fundação Oswaldo Cruz-Fiocruz, Rio de Janeiro, RJ, Brasil; 4 Secretaria Municipal de Saúde, São José do Vale do Rio Preto, RJ, Brasil

**Keywords:** Triatominae, vectors, intradomiciliary infestation, colonisation, Chagas disease

## Abstract

**BACKGROUND:**

*Triatoma vitticeps* (Stål, 1859), a primarily sylvatic vector of *Trypanosoma cruzi* (Chagas, 1909), has been reported invading dwellings and peridomiciliary ecotopes in several regions of Brazil.

**OBJECTIVES and METHODS:**

Here, we report for the first time, the colonisation of a brick house by *T. vitticeps* in the municipality of São José do Vale do Rio Preto, Rio de Janeiro State.

**FINDINGS:**

Inside the house, indicators of colonisation were recorded, including 15 eggs, 11 nymphs, two exuviae, and 19 adult specimens. *T. cruzi* infection was detected in 13 specimens and the blood meal source identification, through amplification of DNA fragments indicated the presence of human blood as one of the feeding sources in seven specimens.

**MAIN CONCLUSIONS:**

The infested dwelling was treated with insecticide to control the infestation, and residents were appropriately instructed to remain vigilant for the presence of triatomines and to contact health authorities if new specimens were detected.

Currently, 159 Triatominae species are recognised as potential vectors of *Trypanosoma cruzi* (Chagas, 1909), the parasite that causes Chagas disease (CD).^1^ ^,2^ The great majority of the vectors are sylvatic, distributed across the Americas and Brazil harbours the highest diversity of triatomine species.^1^ ^,3^ ^,4^

In the State of Rio de Janeiro, *T. cruzi* vectors are primarily sylvatic species associated with the Atlantic Forest. Among these, *Triatoma vitticeps* (Stål, 1859) is a predominantly wild triatomine distributed in the Brazilian states of Bahia, Espírito Santo, Minas Gerais and Rio de Janeiro.^1^ ^,5^ ^,6^ Although primarily associated with forest environments, adults of *T. vitticeps* frequently invade rural dwellings, attracted by artificial light or in search of blood meals.^7^ This synanthropic behaviour links sylvatic transmission cycles to human exposure, particularly in peridomiciliary settings near woodpiles and animal shelters, conferring significant epidemiological relevance to *T. vitticeps* in areas where *T. cruzi* circulates.

The ecology and biology of *T. vitticeps* have been extensively investigated, encompassing aspects of its life cycle, feeding behaviour, defecation patterns, and blood meal sources.^8^ ^,9^ These studies indicate that the relatively long interval between the end of feeding and defecation is one of the main factors limiting the vectorial efficiency of this species.^9^ Nevertheless, blood meal analyses have revealed frequent ingestion of human blood by adults captured indoors, demonstrating direct contact with human populations.^8^ ^,10^

The invasion and the establishment of *T. vitticeps* in anthropogenic environments has been reported by several authors. Peridomiciliary colonies of this species infected with *T. cruzi* were found in rural areas of Espírito Santo State, Brazil.^11^ The population dynamics of that species in Minas Gerais, Brazil showed the infested domiciles were located closer to open fields rather than to the sylvatic environment.^12^ More recently, the ecological and spatial dynamics of this species were modelled and the results pointed out that CD transmission in Espírito Santo is driven by wind-facilitated *T. vitticeps* invasion, with higher mammal biodiversity, predicting increased *T. cruzi* infection rates.^13^

Furthermore, previous records show *T. vitticeps* colonising domicile in the Afredo Chaves municipality, Espírito Santo State, suspected of transmitting CD.^14^ ^,15^ In Rio de Janeiro State, the species was recorded colonising wattle and daub houses, in rural areas, in Miguel Pereira and São Fidelis municipalities.^16^ ^,17^ The present paper reports, for the first time, colonisation by *T. vitticeps* in a brick house located in an urban area adjacent to a severely degraded fragment of the Atlantic Forest in Rio de Janeiro State, Brazil.

## MATERIALS AND METHODS

**Fig. 1:**
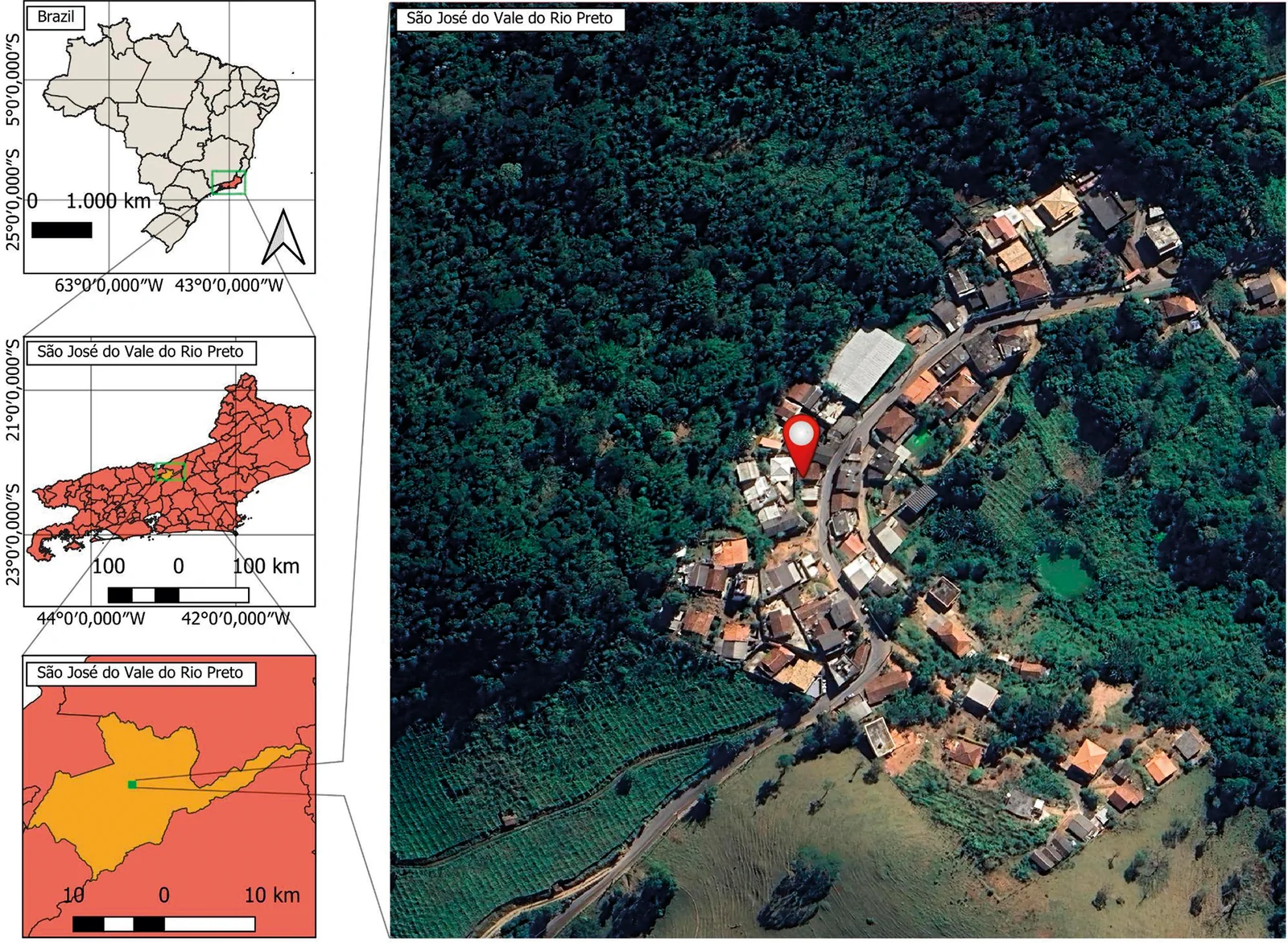
map showing the location of the residence infested by *Triatoma vitticeps*, in São José do Vale do Rio Preto, Rio de Janeiro State, Brazil.

*Study area and specimen collection* - Following a request from the Municipal Health Secretary, the inspected dwelling was in an urbanised area within a heavily degraded fragment of the Atlantic Forest in the municipality of São José do Vale do Rio Preto (SJVRP), Rio de Janeiro State, Brazil, at an altitude of 615 m (22.1600933º S, 42.9361117º W) (Fig. 1). The well finished brick house is situated near several other dwellings constructed with similar materials, including tile roofs, and is surrounded by a peridomiciliary area containing domestic animals such as birds, dogs, and cats. According to residents, opossums are frequently observed in the surroundings, including peridomiciliary areas. Active searches for triatomines in the infested dwelling were systematically conducted in each of its six rooms (one toy room, one bedroom, one living room, one bathroom, a kitchen, and a laundry).

The presence of triatomines was investigated in all potential shelters within the house and in the peridomiciliary areas. The exhaustive sampling effort took 5 h to be completed and was carried out in clockwise direction, in each of the house compartments, plus the roof. All specimens were manually collected using tweezers, placed in plastic containers and subsequently transported to the Laboratório Nacional e Internacional de Referência em Taxonomia de Triatomíneos (LNIRTT), Instituto Oswaldo Cruz. In the laboratory, specimens were identified using the taxonomic keys,^5^ ^,6^ and recorded according to developmental stage, including the number of eggs, exuviae, and male and female adults. One male voucher specimen was deposited under number 20627 in the Triatomine Collection of Instituto Oswaldo Cruz (CTIOC), Rio de Janeiro, Brazil. After the taxonomic identification 16 specimens were selected for analysis.

Due to the relevant risk of the finding for public health, this report was based on a single inspection, regarding the status of colonisation of the captured species in the urban brick residence. Once the searches were carried out, an official technical report was elaborated by LINRTT and sent to the Ministry of Health authorities reporting the results and stressing the importance of implementing vector monitoring activities in SJVRP (Supplementary data). Furthermore, at the end of the exhaustive sampling efforts the residents were properly instructed and requested to leave the house while the spraying treatment was applied for eliminating the infestation.

*DNA extraction* - The DNeasy Blood & Tissue Kit protocol (Qiagen) was used for DNA extraction from the intestinal contents of the specimens. Due to the preservation condition of the specimens received by the laboratory, dried specimens were excluded from molecular analysis, and only engorged insects were selected to ensure greater quantity and quality of DNA in the intestinal content, allowing higher reliability in the identification of the blood meal source. Each specimen was dissected in the terminal region of the abdomen for removal of the intestinal content. The intestinal content was macerated using autoclaved plastic pestles and stored in sterile microcentrifuge tubes at -20ºC until the beginning of molecular analyses.

*Trypanosoma cruzi infection and blood meal source analyses* - To detect natural infection by *T. cruzi* in the captured specimens, the specific primers TcH2AF and TcH2AR were employed. The primers TcH2AF and TcH2AR amplify the histone H2A region. Polymerase chain reaction (PCR) reactions were performed in final volume of 25 μL, using: 12.5 μL Green Taq Polymerase Master Mix (Celco), 0.5 μL of each primer (forward and reverse), 2 μL of DNA, and nuclease-free water to complete the final volume. A fixed volume of 2 μL of intestinal content DNA was used in all PCR reactions. To prevent contamination with human DNA, DNA extraction and PCR procedures were performed in a biosafety cabinet using sterile materials. PCR cycling for the detection of *T. cruzi* was performed under the conditions previously described,^18^ with an initial denaturation at 95ºC for 5 min, followed by 15 cycles of denaturation at 95ºC for 30 s and, extension at 72ºC for 1 min, followed by 30 cycles of denaturation at 95ºC for 30 s, annealing at 60ºC for 30 s, and extension at 72ºC for 30 s, with a final extension at 72ºC for 5 min. To ensure the quality of the DNA present in the samples, primers already described^19^ were used to amplify a fragment of the insect mitochondrial Cytochrome oxidase subunit I (COI).

For blood meal source identification, specific primers^20^ were used for the detection of vertebrate DNA from commonly found animals in domestic environments (birds, dogs, and cats) and humans. The second PCR was carried out for the specific primers with an initial denaturation at 94ºC for 6 min, followed by 40 cycles of 30 s at 94ºC, annealing at 60ºC for 30 s, and extension at 72ºC for 1 min, followed by a final extension at 72ºC for 10 min. The reactions were performed in a gradient thermal cycler and maintained at 12ºC (PR-96E, MIULAB) until collection. PCR products were confirmed by electrophoresis on 1 - 1.5% agarose gels stained with GelRed 20× (Biotium) were run in TBE 1X buffer for 35 min at 90 V. A 100 bp DNA ladder (Ludwig) was used as a molecular weight marker, and gels were visualised under UV light using a transilluminator (Loccus).

**TABLE I:** List of primer sequences for detection of *Trypanosoma cruzi* infection and vertebrate-specific blood meal source used in the analyses of *Triatoma vitticeps* specimens, collected in the intradomiciles in São José do Vale do Rio Preto, Rio de Janeiro, Brazil

Primer	Sequence	Target	Amplicon size (bp)	Reference
TcH2AF	5'-GAGAGTGATCGTGGGAGAGC-3'	*T. cruzi*	234	Paiva et al.^18^
TcH2AR	5'-AGTGGCAGACTTTGG GGTC-3'	*T. cruzi*	234	Paiva et al.^18^
COI (LCO1490)	5'-GGTCAACAAATCATAAAGATATTGG-3'	Triatominae	710	Folmer et al.^19^
COI (HCO2198)	5'-TAAACTTCAGGGTGACCAAAAAATCA-3'	Triatominae	710	Folmer et al.^19^
Human-F	5'-GTAGTACATAAAAACCCAATCCACATC-3'	*Homo sapiens*	470	Ribeiro Jr et al.^20^
Human-R	5'-GTCGGATACAGTTCACTTTAGCTACC-3'	*Homo sapiens*	470	Ribeiro Jr et al.^20^
Avian-F	5'-ATAGAATGGCCTGGGTTGAAAAG-3'	Class avian	197	Ribeiro Jr et al.^20^
Avian-R	5'-AAGTTTTTCACACAGAGGGTGGT-3'	Class avian	197	Ribeiro Jr et al.^20^
Cat -F	5'-ACAGGATCAGAAACCTTTATCTGACTA-3'	*Felis catus*	1179	Ribeiro Jr et al.^20^
Cat-R	5'-TTTGACTTAAAATTTATGGTTTGGTTT-3'	*Felis catus*	1179	Ribeiro Jr et al.^20^
Dog-F	5'-GTCAATGGTTTCAGGACATATAGTTTT-3'	*Canis Familiaris*	476	Ribeiro Jr et al.^20^
Dog-R	5'-TATTGTATGCACTTAGTCCTGTTTTTG-3'	*Canis Familiaris*	476	Ribeiro Jr et al.^20^

Positive controls for *T. cruzi* and vertebrate-specific primers (*Homo sapiens*, *Gallus gallus*, *Felis catus* and *Canis familiaris*) were obtained from samples provided by the Institute of Biology of the State University of Campinas (UNICAMP), and negative controls (distilled water replaced in DNA sample) were included and run on the same gel. Amplicons of 234 bp were interpreted as positive for *T. cruzi*, while amplicons of 470 bp, 476 bp, 1179 bp, and 197 pb were considered positive for human, dog, cat, and bird DNA, respectively (Table I).

## RESULTS

**Fig. 2:**
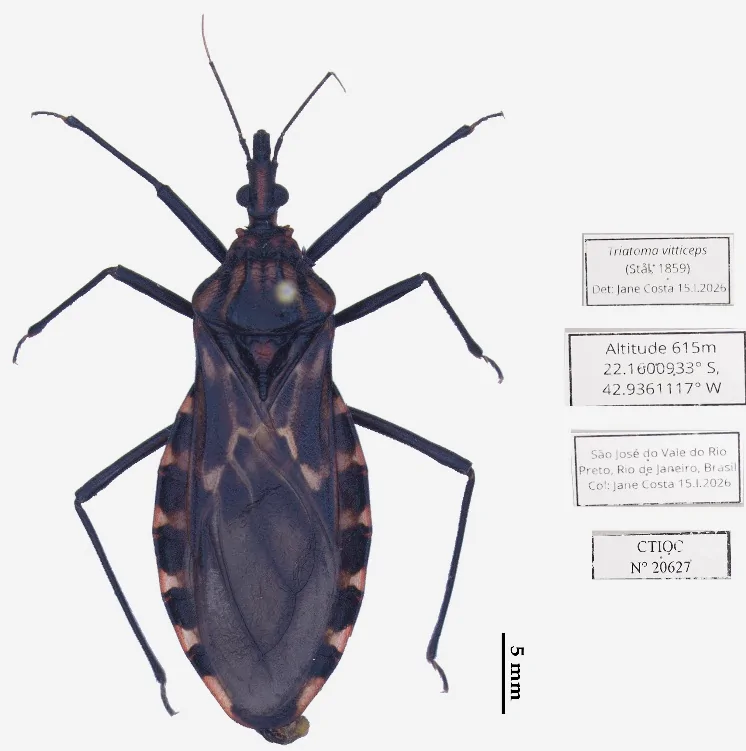
*Triatoma vitticeps* male specimen collected in the intradomicile, in São José do Vale do Rio Preto, Rio de Janeiro State, Brazil, and deposited in the Triatominae Collection of Instituto Oswaldo Cruz.

During a single systematic and exhaustive search covering all rooms, the roof and the peridomiciliary areas of the house, a total of 47 specimens of *T. vitticeps* were found (Table II, Fig. 2).

**TABLE II:** Number of *Triatoma vitticeps* specimens collected in intradomiciliary environment of a brick house in urban area of São José do Vale do Rio Preto municipality, Rio de Janeiro, Brazil

Specimens	Number	Place
Females	9	Toy room
Males	10	Toy room
Nymphs (5th instar)	1	Roof, under the tiles
Nymphs (2th instar)	2	Roof, under the tiles
Nymphs (1th instar)	8	Roof, under the tiles
Eggs	15	Roof, under the tiles
Exuviae	2	Roof, under the tiles
Total	47	

**Fig. 3:**
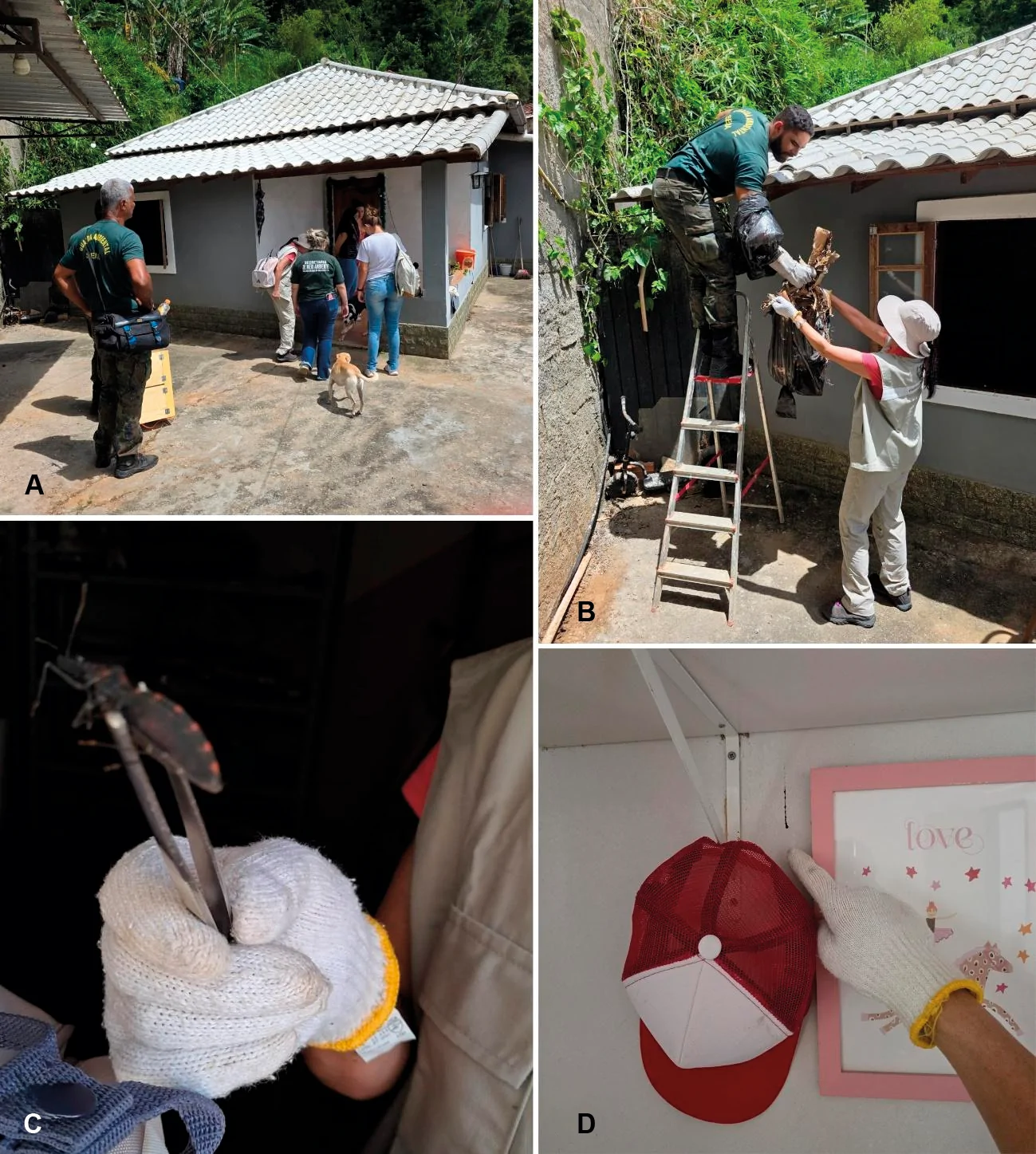
entomological surveillance activities carried out in an urban residential area, in São José do Vale do Rio Preto, Rio de Janeiro State, Brazil. (A) Technical visit to the residence for inspection and guidance to residents. (B) Removal of triatomine specimens and organic materials (feathers, dry leaves, sticks, fur, rodent faeces) accumulated in the shelters on the roof of the dwelling. (C) Manual capture of an adult *Triatoma vitticeps* specimen using entomological tweezer. (D) Inspection of internal structures of the dwelling and observation of spots like triatomine faeces.

**Fig. 4:**
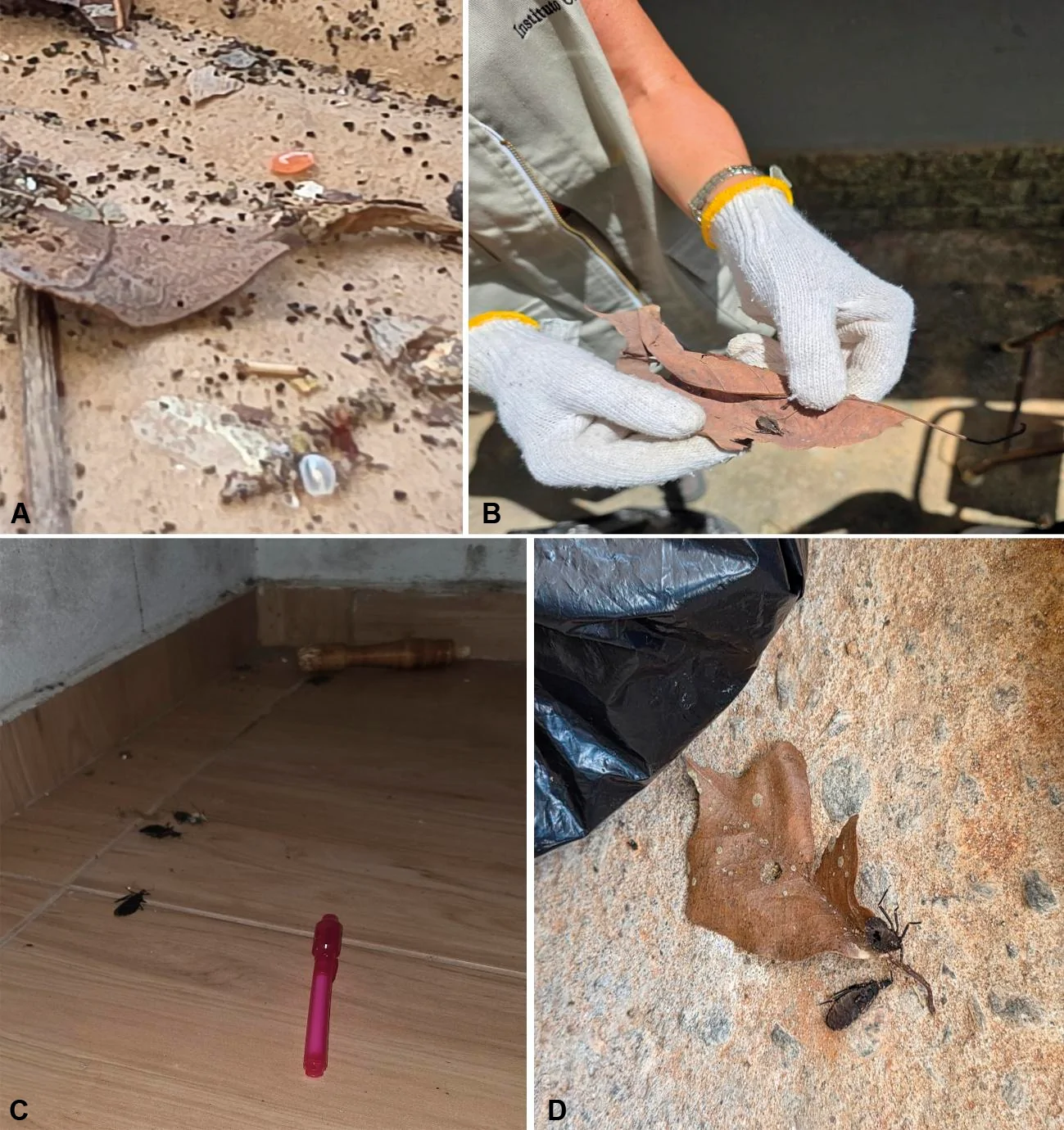
evidences of *Triatoma vitticeps* colonising an urban brick house, in São José do Vale do Rio Preto, Rio de Janeiro State, Brazil. The presence and capture of triatomine bugs in the domiciliary environment: (A) *T. vitticeps* eggs found among the organic material accumulated under the tiles. (B-C) Manual inspection of dry leaves and organic material found under the tiles, serving as triatomine bug shelter, where exuviae were found. (D) Intradomiciliary environment, showing the toy room, where most of the captured triatomine bugs were found.

The adult specimens were found in a single room (the toy room). According to the homeowner, specimens were also frequently observed in the living room and in the bedroom near the bed. Because spots like triatomine faeces were observed on the wall, near the ceiling, in the toy room (where most of the adult specimens were collected), additional searches on the roof were conducted (Fig. 3). Tiles were removed above the area marked with the faecal spots observed indoors. This inspection revealed several dry leaves, bird feathers, fur, sticks and rodent faeces. Among this material, nymphs, exuviae, and eggs of *T. vitticeps* were found (Fig. 4A-D). Other areas of the roof were equally inspected however, no additional signs of triatomine colonisation were found.

From the sixteen specimens tested, thirteen were positive for *T. cruzi* infection. Only three specimens tested negative for *T. cruzi* infection. The expected amplification of the COI gene fragment was observed in fifteen specimens. Blood meal source identification, based on the amplification of DNA fragments, indicated the presence of human blood as one of the feeding sources, with seven triatomine specimens testing positive. None of the specimens tested positive for dog or cat DNA. One specimen presented a mixed blood meal, with human and bird DNA detected; this specimen was also positive for *T. cruzi* infection.

## DISCUSSION

Over recent decades, Latin America has observed a notable increase in new records of triatomine species occurrences. Those belonging to the genera *Triatoma* and *Panstrongylus* are the most frequently detected in human dwellings and exhibit high rates of *T. cruzi* infection. Infected triatomine species have been recorded across a broad geographic range, from Argentina to the United States. Certain species have become increasingly intrusive, repeatedly invading dwellings and, in some instances, establishing stable colonies in urban environments, leading to a rise in reported encounters.^4^ ^,21^ ^,22^ ^,23^ This trend is exemplified by the recent first records of *Panstrongylus geniculatus* (Latreille 1811) and *Panstrongylus megistus* (Burmeister 1835) on Ilha Grande, an Atlantic Forest island, illustrating the intrusive behaviour of native triatomines into anthropic environments.^24^ ^,25^ These findings highlight the ongoing geographic expansion and adaptive potential of these vectors, underscoring the need for enhanced urban and peri-urban surveillance to mitigate CD transmission risk amid changing ecological and anthropogenic landscapes.^26^ ^,27^ ^,28^

Endemic from Brazil and considered primarily invasive, *T. vitticeps* has been recorded invading houses attracted by light from nearby forest remnants in the Atlantic Forest biome.^5^ ^,6^ As previously mentioned, records of *T. vitticeps* colonising domiciles and peridomiciles in rural areas were reported in literature.^11^ ^,12^ ^,16^ ^,17^ The colonisation of anthropic ecotopes by *T. vitticeps* in Alfredo Chaves was reported and the authors emphasised there are no indications of change in the sylvatic habits of that species, suggesting the colonies found were associated with opossum nests, a result of an expansion of the sylvatic environment into the peridomicile.^11^

Additional studies have confirmed the presence of both nymphs, indicating active breeding, and adults inside houses and associated outbuildings, particularly in areas of Rio de Janeiro, Minas Gerais and Espirito Santo, where the species is frequently associated with opossum nests (*Didelphis aurita*).^7^ ^,9^ ^,16^ ^,29^ Despite the fact *T. vitticeps* presents small domiciliary colonies, these records suggest a broader ecological plasticity than previously recognised for this vector species, raising concerns in terms of public health.

The findings here reported confirm the colonisation capacity of *T. vitticeps* since eggs, nymphs, adults and exuviae were found in the intradomiciliary area. The previously published studies on *T. vitticeps* showing the species colonising wattle and daub houses in rural areas, and infection by *T. cruzi* in anthropogenic rural settings demonstrate our results are not unprecedented. However, the present report reveals the inedited finding of *T. vitticeps* colonising a brick house of an urban area, in SJVRP. It is also worth mentioning that according to the local health authorities, there are no previous records of *T. vitticeps* occurring in the referred municipality.

The presence of eggs, nymphs, exuviae and adults of *T. vitticeps* in intradomiciliary environment provide clear evidence the species was colonising an urban brick house. Nevertheless, the finding was based on one single sampling event. Due to the public health risk it represents, the infested house was sprayed with insecticide the day after the exhaustive triatomine capture was carried out. In order to get a broad understanding of a possible adaptation process of *T. vitticeps* to anthropic ecotopes further studies must be conducted in the mentioned municipality.

*Triatoma vitticeps* specimens captured inside dwellings have consistently shown high rates of natural infection with *T. cruzi*.^9^ The analysed specimens in this report also confirm *T. vitticeps* as a highly infected vector, since from 16 tested specimens, 13 were positive for *T. cruzi*, and despite these high infection rates with the etiologic agent of CD and other trypanosomes, *T. vitticeps* has traditionally been classified as a secondary vector, mainly due to its low tendency to colonise houses and the long interval between hematophagy and defecation, which reduces the efficiency of classical vectorial transmission.^9^ However, this species has been shown to harbour single and mixed infections involving multiple *T. cruzi* discrete typing units (TcI-TcIV), as well as *T. cruzi marinkellei* and *T. dionisii*. Importantly, fatal cases of acute CD associated with oral transmission following the manipulation of infected *T. vitticeps* specimens have been documented.^30^

Because the recognition of an anthropophilic triatomine is also key to define a relevant CD vector in the classical *T. cruzi* transmission, this characteristic has been evaluated for several species.^31^ For *T. vitticeps*, results were obtained through the precipitin test^8^ ^,10^ ^,16^ demonstrating the studied species presents a significant feeding eclecticism and marked anthropophily. In the present study, anthropophily was evaluated using specific primers for vertebrate DNA detection of commonly found animals in domestic environments like birds, dogs, cats, and humans.^20^ From the 13 tested specimens, seven were positive for human blood, stressing the importance to constantly monitor the area studied for triatomine infestation in the domiciliary ecotopes.

The present report highlights the importance of advising residents to carefully collect and deliver suspected "kissing bugs" (barbeiros) to local health services for identification and testing, rather than crushing them, as was recommended to inhabitants of the neighbourhood where the infestation was detected. In addition, evaluating environmental changes that may be facilitating triatomine invasions in the study area is essential for implementing proactive measures to prevent new infestations.^13^ ^,27^ ^,32^ ^,33^

The presence of *T. vitticeps* (eggs, adults and nymphs) in the domiciliary environment of a brick house reinforces the need for sustained entomological surveillance. Emerging evidence also suggests that what is currently identified as *T. vitticeps* may represent a complex of cryptic species, which could help explain observed variations in behaviour, infection rates, and patterns of domiciliary invasion.^34^ Also, a detailed study on the *T. vitticeps* phenotype, encompassing specimens from a broad range of its geographic distribution, is recommended since distinct phenotypes may present distinct epidemiological importance requiring distinct control strategies.^35^ Furthermore, detailed studies of the *T. cruzi* lineages and the food sources of the mentioned vector are necessary.

After the entomological survey, local inhabitants were instructed to promptly contact health authorities whenever suspected insects are found in their homes or surrounding areas. In this context, digital surveillance and citizen science initiatives such as WhatsBarb tool,^36^ and the CD Portal (https://chagas.fiocruz.br/), launched by the Oswaldo Cruz Foundation (Fiocruz), Brazil, are crucial to disseminate knowledge about CD. These initiatives facilitate early detection, rapid communication with surveillance services, and proper taxonomic identification of triatomines, strengthening entomological surveillance in areas where new infestations are detected.^1^ ^,4^

In order to check in details and evaluate the results of the insecticide spraying in the infested brick house here reported and, in the neighbourhood, future searches are programmed in collaboration with the technicians of the local Health Secretary. A research project is going to be implemented to provide a comprehensive and integrative analysis allowing the understanding of the *T. cruzi* cycles occurring in the infested area, as carried out by our team for other vector species.^31^ ^,37^

*In conclusion* - Here we report, for the first time, a triatomine colonisation in SJVRP, by *T. vitticeps*, a species primarily considered a sylvatic vector. Specimens were found infesting a brick house, representing a new and unexpected event recorded in a highly degraded fragment of the Atlantic Forest, in an urban area. Laboratory analyses showed *T. vitticeps* specimens were infected by *T. cruzi* and reacted positively for human blood as a food source, confirming public health risks.

Integrative studies, including all aspects of the *T. cruzi* transmission cycle are required to understand the eco-epidemiological dynamics of CD in SJVRP. The mentioned endeavours are imperative to support proactive measures aimed at preventing human infection. In addition, continuous educational campaigns are crucial to raise awareness among the locals.

## SUPPLEMENTARY MATERIALS

Supplementary material

## Data Availability

The contents underlying the research text are included in the manuscript.
