# Supplementary material for: First record of Triatoma vitticeps (Hemiptera, Reduviidae) colonising a brick house in an urban area of a devastated Atlantic Forest fragment in Rio de Janeiro State, Brazil
Source: Mem Inst Oswaldo Cruz. 2026 Aug 31;121:e260038. doi: 10.1590/0074-02760260038 (PMC13532567; doi:10.1590/0074-02760260038)
Supplement: Supplementary file 1 [file 1678-8060-mioc-121-e260038-s1.pdf]

FIOCRUZ

Instituto Oswaldo Cruz
Laboratório Nacional e Internacional de Referência em Taxonomia de Triatomíneos

NOTA TÉCNICA Nº 01/2026/LNIRTT/IOC/FIOCRUZ

Assunto: Registro de colonização domiciliar por espécie nativa de triatomíneo (*Triatoma vitticeps*) no município de São José do Vale do Rio Preto, estado do Rio de Janeiro.

Interessados: Secretaria Municipal de Saúde; Secretaria do Meio Ambiente; Vigilância em Saúde; Vigilância Epidemiológica; Vigilância Entomológica; Secretaria de Vigilância em Saúde e Ambiente (SVSA) do MS.

1. CONTEXTUALIZAÇÃO

O Laboratório Nacional e Internacional de Referência em Taxonomia de Triatomíneos (LNIRTT), do Instituto Oswaldo Cruz (IOC/FIOCRUZ), no uso de suas atribuições como laboratório de referência, vem, por meio desta, comunicar o **registro de colonização domiciliar por triatomíneos da espécie *Triatoma vitticeps***, composta por **ovos, ninfas e adultos**, **com detecção de infecção natural por *Trypanosoma cruzi* em treze espécimes e evidência de alimentação em sangue humano em sete espécimes analisados**, em domicílio localizado no município de São José do Vale do Rio Preto, estado do Rio de Janeiro.

A espécie *Triatoma vitticeps* é considerada nativa da Mata Atlântica e, historicamente, associada a ambientes silvestres. Entretanto, registros de invasão e colonização de ambientes domiciliares e peridomiciliares têm sido descritos na literatura, o que representa potencial risco à saúde pública, especialmente em áreas com circulação do parasito. Registros como este reforçam seu potencial de adaptação ao ambiente domiciliar, especialmente em áreas com alterações ambientais, demandando atenção contínua da vigilância entomológica e epidemiológica.

2. ANÁLISE DO RISCO

A constatação de colonização domiciliar, associada à infecção natural por *T. cruzi* e à evidência de alimentação em sangue humano, sugere interação direta entre os triatomíneos e moradores do domicílio, configurando situação de risco epidemiológico para transmissão vetorial da doença de Chagas.

3. RECOMENDAÇÕES

Diante do exposto, recomenda-se a intensificação das ações de vigilância e controle, com especial atenção às seguintes medidas:

Medidas de orientação voltadas aos moradores:

Melhoria e manejo do ambiente domiciliar e peridomiciliar:
Realizar o reboco e tamponamento de rachaduras, frestas e buracos em paredes, pisos e tetos;
Instalar telas (tipo mosquiteiras) em portas e janelas;
Evitar o acúmulo de lenha, telhas, entulhos ou materiais diversos no interior e nos arredores das residências;
Retirar ninhos de aves e mamíferos silvestres dos beirais e telhados das casas;
Manter limpeza periódica das residências e quintais;
Evitar construções muito próximas a áreas de mata;
Utilizar mosquiteiros sobre as camas;
Evitar o uso de lâmpadas acesas durante a noite próximas aos dormitórios, pois a luz pode atrair os insetos;
Em caso de presença de galinheiros, chiqueiros, depósitos e outras estruturas, mantê-los afastados das casas e sempre limpos.
Utilizar roupas de mangas compridas e calças durante a noite, especialmente em áreas rurais ou periurbanas.

Medidas para prevenção da transmissão vetorial-oral:
Lavar cuidadosamente frutas, legumes e verduras antes do consumo;
Manter os alimentos armazenados em recipientes fechados;

Lavar adequadamente equipamentos utilizados no preparo de alimentos *in natura* (liquidificadores, moendas de cana-de-açúcar ou outros sucos, batedores de açaí, etc.) antes e após o uso;
No caso do consumo de açaí ou outros produtos artesanais, verificar se o estabelecimento possui certificações e segue as normas sanitárias vigentes.

Em caso de dúvidas, buscar orientação junto à Vigilância Sanitária Municipal.

4. ENCAMINHAMENTOS

Recomenda-se que as autoridades municipais de saúde:
Reforcem as ações de busca ativa e notificação de triatomíneos;
Intensifiquem as atividades educativas junto à população;
Avaliem a necessidade de investigação epidemiológica complementar.
Articulem-se com os serviços estaduais e federais de vigilância em saúde.

Recomenda-se que a Secretaria Municipal de Saúde, por meio da Vigilância Epidemiológica e da Atenção Primária à Saúde, avalie a necessidade de acompanhamento clínico dos moradores do domicílio afetado, considerando o risco epidemiológico associado à presença de colônia domiciliar de *Triatoma vitticeps*.

Caso julgado pertinente pela autoridade sanitária local, orienta-se:

A realização de avaliação clínica inicial na rede de Atenção Básica, conforme os fluxos assistenciais do Sistema Único de Saúde (SUS);

A **i**ndicação de exames laboratoriais para doença de Chagas, de acordo com os protocolos vigentes do Ministério da Saúde;

Recomenda-se, ainda, que a Secretaria Estadual de Saúde seja comunicada para apoio técnico e, se necessário, para a articulação com serviços de referência estaduais.

O Ministério da Saúde deverá ser informado por meio dos fluxos regulares de vigilância, conforme normativas vigentes, para fins de monitoramento epidemiológico e apoio técnico

O Serviço de Referência em Taxonomia de Triatomíneos do LNIRTT/IOC/FIOCRUZ permanece à disposição para apoio técnico e demais esclarecimentos que se fizerem necessários.

Rio de Janeiro, 8 de fevereiro de 2026.

Chefe do LNIRTT, Instituto Oswaldo Cruz, FIOCRUZ

Av. Brasil 4365, Pavilhão Rocha Lima 5º andar, Caixa Postal 926, 21040-900,
Rio de Janeiro, RJ, Brasil
Tel.: (+ 55 21) 25621621

The official technical report elaborated by Laboratório Nacional e Internacional de Referência em Taxonomia de Triatomíneos (LNIRTT) and sent to the Ministry of Health authorities reporting the results and stressing the importance of implementing vector monitoring activities in the municipality of São José do Vale do Rio Preto.

SUPPLEMENTARY DATA
