# Supplementary material for: First record of Triatoma vitticeps (Hemiptera, Reduviidae) colonising a brick house in an urban area of a devastated Atlantic Forest fragment in Rio de Janeiro State, Brazil
Source: Mem Inst Oswaldo Cruz. 2026 Aug 31;121:e260038. doi: 10.1590/0074-02760260038 (PMC13532567; doi:10.1590/0074-02760260038)
Supplement: Peer review history

# Reviewer report: FIRST REVIEW ROUND - REVIEWERS COMMENTS

## REVIEWER #1

The manuscript presents a relevant and potentially publishable record, particularly by documenting the intradomiciliary colonization of *Triatoma vitticeps* in an urban environment, which carries important epidemiological implications. However, despite its merits, the study exhibits conceptual, methodological, and, most notably, interpretative weaknesses that should be addressed to enhance its scientific robustness and to avoid extrapolations that may be questioned during peer review.

Overall, the manuscript is well structured and clearly written. Nevertheless, there is a recurring tendency to overemphasize the findings without sufficient analytical support. The title is strong and appropriate; however, the claim of a "first record" requires more rigorous substantiation. It should be clearly specified whether this refers to the first record for the municipality, for a particular type of urban construction, or for a fully documented domiciliary colonization including all developmental stages. This distinction is essential, as previous records of domiciliary colonization by *T. vitticeps* exist in southeastern Brazil, and the originality of the study may be questioned if its scope is not precisely defined.

In this context, it is crucial to incorporate existing literature demonstrating colonization or establishment of this species in anthropogenic environments. Relevant studies include: Peridomiciliary colonies of *Triatoma vitticeps* (Stål, 1859) (Hemiptera, Reduviidae, Triatominae) infected with *Trypanosoma cruzi* in rural areas of the state of Espírito Santo, Brazil (Biral dos Santos et al., Memórias do Instituto Oswaldo Cruz 100(5): 471–473, 2005); Population dynamics of *Triatoma vitticeps* (Stål, 1859) in Itanhomi, Minas Gerais, Brazil (de Souza et al., Memórias do Instituto Oswaldo Cruz 103(1): 14–20, 2008); and Unveiling the ecology and spatial dynamics of *Trypanosoma cruzi*, its DTUs and *Triatoma vitticeps* in the Atlantic Forest of south-eastern Espírito Santo State, Brazil (Testai et al., PLoS Neglected Tropical Diseases 20(3): e0014111, 2026). These studies demonstrate that colonization and infection in anthropogenic settings are not entirely unprecedented, even if the specific context presented here may be.

The abstract is clear but would benefit from greater quantitative precision. The expression "several indicators of colonization" should be replaced with objective data, such as the number of nymphs, eggs, and adults observed. Additionally, the statement regarding *Trypanosoma cruzi* infection should avoid vague wording such as "presented and discussed" and instead include the key findings, even in summarized form, to strengthen its impact.

The introduction is generally well contextualized but contains some redundancies and overly broad generalizations. While the discussion of synanthropic behavior and epidemiological relevance is appropriate, it should be more directly aligned with the specific knowledge gap addressed by the study. The central objective remains somewhat unclear whether the study aims to demonstrate colonization, assess epidemiological risk, or discuss urban adaptation. At present, these aspects are addressed simultaneously, which dilutes the focus. Incorporating overlooked studies directly addressing the ecology and domiciliation of *T. vitticeps* including those cited above would significantly improve the contextual framework.

The methodology presents several critical issues. Although the collection procedures are adequately described, there is no standardization of sampling effort. The study is based on a single inspection, which substantially limits inferences regarding stable colonization, and this limitation should be explicitly acknowledged. The selection of only 16 adults for molecular analysis also requires clearer justification, particularly given that 47 specimens were collected. Furthermore, restricting the analysis to engorged insects reduces representativeness and may introduce bias in the identification of blood meal sources.

The molecular section is functional but lacks detail expected by current standards. There is no information regarding the use of positive and negative controls for each primer in the blood meal analyses, nor is there discussion of potential cross-amplification. The statement that results were "adopted due to the rapidity and high quality of the analyses" is not appropriate for a scientific manuscript and should be removed or reformulated based on technical criteria.

In the results section, a major concern is the classification of 13 out of 16 specimens as "possibly positive" for *T. cruzi*. In PCR-based analyses, results should be clearly defined as positive or negative according to objective criteria. The use of "possibly" undermines data reliability and raises methodological concerns. This issue should be clarified, whether in terms of band intensity, reproducibility, or predefined thresholds for positivity. Without such clarification, the validity of the results may be questioned.

Additionally, the results could be presented in a more quantitative and structured manner. For example, the spatial distribution of insects within the household could be better organized in a table or described more objectively. Table 1 also requires revision to correct typographical errors and ensure consistent formatting.

The discussion represents the most sensitive section of the manuscript. Although it provides useful context, it relies on excessive extrapolation from a single sampling event. The text suggests broader changes in epidemiological dynamics and urban adaptation; however, the data are limited to a single household and a single collection event. The tone should therefore be moderated, clearly framing the findings as a punctual yet relevant record. In this regard, incorporating additional literature is essential to support and calibrate the interpretations. The recent study by Testai et al. (2026) is particularly relevant, as it offers an integrated perspective on vector and parasite ecology in the Atlantic Forest and could strengthen the discussion of infection patterns, parasite diversity, and spatial dynamics.

The discussion of oral transmission, although conceptually valid, appears somewhat disconnected from the data presented and may be perceived as overstating the study's implications. It would be more appropriate to maintain focus on domiciliary colonization and vector–human contact, as supported by the blood meal analysis.

The section addressing anthropophily and blood meal sources is interesting but should also be interpreted with caution. The detection of human blood in seven individuals within a small sample size does not support robust conclusions about population-level feeding behavior. This finding should be presented as indicative rather than conclusive, ideally in comparison with previous studies demonstrating the species' eclectic feeding habits.

A notable strength of the study is the identification of a colony under the roof, including eggs, nymphs, and exuviae, which provides clear evidence of colonization. This represents the core contribution of the manuscript and should be emphasized further, including direct comparisons with similar records reported in peridomiciliary environments in southeastern Brazil.

It is also strongly recommended that the authors include a high-quality dorsal photograph of at least one collected specimen. This would allow proper documentation of the morphotype, support taxonomic identification, and enhance the overall robustness and transparency of the study.

The final portion of the discussion combines recommendations, future perspectives, and outreach aspects in a somewhat diffuse manner. The mention of tools such as WhatsBarb is interesting but appears tangential to the main scientific argument and could be presented more concisely.

The conclusions are coherent but partially repetitive and employ somewhat strong language given the level of evidence. They should be made more objective and strictly aligned with the data presented.

From a technical standpoint, there are several minor issues throughout the manuscript, including inconsistencies in species nomenclature, errors in figure legends, and problems in references and formatting. Although these do not compromise the scientific content, they should be carefully revised, as they affect the overall perception of quality.

In summary, the manuscript has clear merit and publication potential, particularly due to the record of intradomiciliary colonization in an urban setting. However, it requires substantial revisions, including more rigorous data interpretation, clearer methodological descriptions (especially regarding PCR procedures), reduction of unsupported extrapolations, improved data presentation, inclusion of a dorsal image documenting the specimen, and incorporation of relevant overlooked literature. Addressing these points will significantly strengthen the manuscript and reduce the likelihood of predictable reviewer criticisms.

## REVIEWER #2

Thank you for presenting this detailed assessment. I have some comments to help with clarity but overall, excellent presentation.

- Please comment on exactly why the Municipal Health Secretary was requested to the specific home? Was this because a resident at the home reconginzed a triatomine? Where they being bitten by teh bug? Chagas disease concerns? Please be more specific and include in "Study area and specimen collection" section.

- How long was the investigation? Was it for one day? 8 hours? Did they return for multiple entomological assessments? Please put in the details of the investigation timeline.

- Figure 2 (D) shows fecal drop on the walls but how was this confirmed? Some insects will defecate on walls and it looks like triatomine feces. I suggest saying, "Inspection of internal structures of the dwelling and observation of suspected triatomine feces on the wall."

- Did you test for DTU I? Why not?

- Please provide more details of the blood meal analysis. This is very briefly mentioned. Human blood was detected but documenting the other sources aids in understanding the extent to the other blood meal sources and will also help with the ecology of domestic invasion of a triatomine like this.

- I like the pictures of the triatomines and where they were found. This is impactful. I found myself wanting to see at least one adult picture which shows clear morphological features of the bug. This would also be impactful for readers.

- Map is excellent. Fantastic!

- Please add in the Discussion the known prevalence of Chagas in this locality and in the Rio de Janeiro State.

- I would also add some sentences on the impact of a sylvatic vector invading into the peridomestic domain and now domicilatting a home. Why did this occur at this residence? As you mentioned this is a fragmented forest region and deforestation is being seen in many regions of Brasil and other regions where Chagas disease is endemic. Sylvatic and peridomestic vectors are now being seen more and more in these regions. I would make some comments about this and possible site some article displaying this:

- Cantillo-Barraza O, Gual-González L, Velásquez-Ortiz N, Medina Camargo MA, González P, Cruz-Saavedra L, Castillo A, Zuluaga S, Herrera G, Cowan H, Velez-Mira A, Patiño LH, Ramírez JD, Triana O, Nolan MS. Triatoma venosa and Panstrongylus geniculatus challenge the certification of interruption of vectorial *Trypanosoma cruzi* transmission by Rhodnius prolixus in eastern Colombia. PLoS Negl Trop Dis. 2025 Jan 27;19(1):e0012822.

- Meraz-Medina T, Vences-Blanco MO, Ramírez-Cervantes AG, Nogueda-Torres B, Martínez-Ibarra JA. First record and vital statistics of Triatoma longipennis (Heteroptera, Reduviidae, Triatominae) from Southern Mexico. Acta Trop. 2025 Nov;271:107873.

- Beatty NL, Bhosale CR, White ZS, Torhorst CW, Wilson KN, Dorleans R, Stenn TMS, Killets KC, Curtis-Robles R, Burkett-Cadena N, Nováková E, Hamer GL, Hamer SA, Wisely SM. Field evidence of *Trypanosoma cruzi* infection, diverse host use and invasion of human dwellings by the Chagas disease vector in Florida, USA. PLoS Negl Trop Dis. 2025 Jul 7;19(7):e0012920.

Other examples exist. I recommend discussing the importance a bit more as it is a great example of the changing dynamics of Chagas disease within the Americas.

Thank you for your work!

## AUTHORS' RESPONSE TO THE REVIEWERS

Dear Editors,

We have forwarded the document with the responses to the reviewers.

Thank you for your contributions, which improved the manuscript. All changes have been considered and highlighted.

Sincerely,

The authors

**Reviewer: 1**

R1: The manuscript presents a relevant and potentially publishable record, particularly by documenting the intradomiciliary colonization of *Triatoma vitticeps* in an urban environment, which carries important epidemiological implications. However, despite its merits, the study exhibits conceptual, methodological, and, most notably, interpretative weaknesses that should be addressed to enhance its scientific robustness and to avoid extrapolations that may be questioned during peer review.

*A:* Dear Reviewer, we thank the valuable comments, suggestions and corrections enhancing the scientific robustness of our manuscript. Bellow we answer each mentioned point one by one.

R1: Overall, the manuscript is well structured and clearly written. Nevertheless, there is a recurring tendency to overemphasize the findings without sufficient analytical support. The title is strong and appropriate; however, the claim of a "first record" requires more rigorous substantiation. It should be clearly specified whether this refers to the first record for the municipality, for a particular type of urban construction, or for a fully documented domiciliary colonization including all developmental stages. This distinction is essential, as previous records of domiciliary colonization by *T. vitticeps* exist in southeastern Brazil, and the originality of the study may be questioned if its scope is not precisely defined.

*A:* Thanks for bringing more clarity for this point. A few lines stressing the first record refers to the encounter of triatomines in a brick house were added. However, previous colonization process of *T. vitticeps* had already been found in a wattle and daub house in another near municipality. This point was also added in the manuscript. Besides that we stressed in the discussion, that according to the Municipal Health Secretary, no previous records of triatomines invading or colonizing houses were found either in the files of the Secretary of Heath of SJV Rio Preto or in the literature.

R1: In this context, it is crucial to incorporate existing literature demonstrating colonization or establishment of this species in anthropogenic environments. Relevant studies include: Peridomiciliary colonies of *Triatoma vitticeps* (Stål, 1859) (Hemiptera, Reduviidae, Triatominae) infected with *Trypanosoma cruzi* in rural areas of the state of Espírito Santo, Brazil (Biral dos Santos et al., Memórias do Instituto Oswaldo Cruz 100(5): 471–473, 2005); Population dynamics of *Triatoma vitticeps* (Stål, 1859) in Itanhomi, Minas Gerais, Brazil (de Souza et al., Memórias do Instituto Oswaldo Cruz 103(1): 14–20, 2008); and Unveiling the ecology and spatial dynamics of *Trypanosoma cruzi*, its DTUs and *Triatoma vitticeps* in the Atlantic Forest of south-eastern Espírito Santo State, Brazil (Testai et al., PLoS Neglected Tropical Diseases 20(3): e0014111, 2026). These studies demonstrate that colonization and infection in anthropogenic settings are not entirely unprecedented, even if the specific context presented here may be.

*A:* We included all suggested references and a few lines mentioning the previous records of *T. vitticeps* colonizing in anthropic environments in rural areas of Espirito Santo and Minas Gerais. The species had already been recorded colonizing wattle and daub houses, in rural areas, in Miguel Pereira and São Fidelis municipalities, in Rio de Janeiro State, (Lorosa et al 2003 e 2008, respectively). All these points were emphasized in the introduction.

R1: The abstract is clear but would benefit from greater quantitative precision. The expression "several indicators of colonization" should be replaced with objective data, such as the number of nymphs, eggs, and adults observed. Additionally, the statement regarding *Trypanosoma cruzi* infection should avoid vague wording such as "presented and discussed" and instead include the key findings, even in summarized form, to strengthen its impact.

*A:* Precise information was included in the abstract and vague wording was changed by objective data.

R1: The introduction is generally well contextualized but contains some redundancies and overly broad generalizations. While the discussion of synanthropic behavior and epidemiological relevance is appropriate, it should be more directly aligned with the specific knowledge gap addressed by the study. The central objective remains somewhat unclear whether the study aims to demonstrate colonization, assess epidemiological risk, or discuss urban adaptation. At present, these aspects are addressed simultaneously, which dilutes the focus. Incorporating overlooked studies directly addressing the ecology and domiciliation of *T. vitticeps* including those cited above would significantly improve the contextual framework.

*A:* The introduction was modified stressing the demonstration of *T. vitticeps* collonization in rural areas in ES and MG, mentioning the previous records of colonization in wattle and daub houses in two other municipalities of RJ. In a near future, we plan to bring more details on ecology and epidemiological risks in the studied area. As recommended, we also added additional references in the discussion and mentioned separately the colonization and urban adaptation.

R1: The methodology presents several critical issues. Although the collection procedures are adequately described, there is no standardization of sampling effort. The study is based on a single inspection, which substantially limits inferences regarding stable colonization, and this limitation should be explicitly acknowledged. The selection of only 16 adults for molecular analysis also requires clearer justification, particularly given that 47 specimens were collected. Furthermore, restricting the analysis to engorged insects reduces representativeness and may introduce bias in the identification of blood meal sources.

*A:* Due to the relevance of the finding and the need to carry out control activities in the infested domicile we decided to focus in the protection of the residents rather than to extend the sampling efforts. That's why just one search for insects was carried out. All the mentioned points were included the M&M and the official technical report elaborated by LINIRTT was included in the M&M as supplementary material.

Besides that, the results here presented are going to be crucial to support further studies, which are going to apply refined molecular methods to define the feeding sources of *T. vitticeps* in the area.

R1: The molecular section is functional but lacks detail expected by current standards. There is no information regarding the use of positive and negative controls for each primer in the blood meal analyses, nor is there discussion of potential cross-amplification. The statement that results were "adopted due to the rapidity and high quality of the analyses" is not appropriate for a scientific manuscript and should be removed or reformulated based on technical criteria.

*A:* More detailed and appropriate information on the molecular data was included in the manuscript.

R1: In the results section, a major concern is the classification of 13 out of 16 specimens as "possibly positive" for *T. cruzi*. In PCR-based analyses, results should be clearly defined as positive or negative according to objective criteria. The use of "possibly" undermines data reliability and raises methodological concerns. This issue should be clarified, whether in terms of band intensity, reproducibility, or predefined thresholds for positivity. Without such clarification, the validity of the results may be questioned.

*A:* The text was modified and the points mentioned were clarified and precisely presented in the results section.

R1: Additionally, the results could be presented in a more quantitative and structured manner. For example, the spatial distribution of insects within the household could be better organized in a table or described more objectively. Table 1 also requires revision to correct typographical errors and ensure consistent formatting.

*A:* Detailed results on spatial distribution of the insects in the house were added in the manuscript and the Table 1 was revised.

R1: The discussion represents the most sensitive section of the manuscript. Although it provides useful context, it relies on excessive extrapolation from a single sampling event. The text suggests broader changes in epidemiological dynamics and urban adaptation; however, the data are limited to a single household and a single collection event. The tone should therefore be moderated, clearly framing the findings as a punctual yet relevant record. In this regard, incorporating additional literature is essential to support and calibrate the interpretations. The recent study by Testai et al. (2026) is particularly relevant, as it offers an integrated perspective on vector and parasite ecology in the Atlantic Forest and could strengthen the discussion of infection patterns, parasite diversity, and spatial dynamics.

*A:* The tone about the infestation was moderated in the discussion, clearly framing the findings as a punctual yet relevant record. We also incorporated additional literature supporting and calibrating the interpretations.

R1: The discussion of oral transmission, although conceptually valid, appears somewhat disconnected from the data presented and may be perceived as overstating the study's implications. It would be more appropriate to maintain focus on domiciliary colonization and vector–human contact, as supported by the blood meal analysis.

*A:* We removed oral transmission paragraph.

R1: The section addressing anthropophily and blood meal sources is interesting but should also be interpreted with caution. The detection of human blood in seven individuals within a small sample size does not support robust conclusions about population-level feeding behavior. This finding should be presented as indicative rather than conclusive, ideally in comparison with previous studies demonstrating the species' eclectic feeding habits.

*A:* We agree with the considerations and the detection of human blood was presented as indicative rather than conclusive, and comparisons with previous studies demonstrating the species' eclectic feeding habits were included.

R1: A notable strength of the study is the identification of a colony under the roof, including eggs, nymphs, and exuviae, which provides clear evidence of colonization. This represents the core contribution of the manuscript and should be emphasized further, including direct comparisons with similar records reported in peridomiciliary environments in southeastern Brazil.

*A:* We agree and the colony found under the roof was emphasized and compared with similar records reported in peridomiciliary environments.

R1: It is also strongly recommended that the authors include a high-quality dorsal photograph of at least one collected specimen. This would allow proper documentation of the morphotype, support taxonomic identification, and enhance the overall robustness and transparency of the study.

*A:* A high-quality dorsal photograph was included in the manuscript.

R1: The final portion of the discussion combines recommendations, future perspectives, and outreach aspects in a somewhat diffuse manner. The mention of tools such as WhatsBarb is interesting but appears tangential to the main scientific argument and could be presented more concisely.

*A:* The final portion of the manuscript was reduced and the WhatsBarb was presented concisely.

R1: The conclusions are coherent but partially repetitive and employ somewhat strong language given the level of evidence. They should be made more objective and strictly aligned with the data presented.

*A:* We changed the conclusion making it more objective and strictly aligned with the results.

R1: From a technical standpoint, there are several minor issues throughout the manuscript, including inconsistencies in species nomenclature, errors in figure legends, and problems in references and formatting. Although these do not compromise the scientific content, they should be carefully revised, as they affect the overall perception of quality.

*A:* We have corrected the inconsistencies in species nomenclature, legends and references formatting.

R1: In summary, the manuscript has clear merit and publication potential, particularly due to the record of intradomiciliary colonization in an urban setting. However, it requires substantial revisions, including more rigorous data interpretation, clearer methodological descriptions (especially regarding PCR procedures), reduction of unsupported extrapolations, improved data presentation, inclusion of a dorsal image documenting the specimen, and incorporation of relevant overlooked literature. Addressing these points will significantly strengthen the manuscript and reduce the likelihood of predictable reviewer criticisms.

*A:* Thanks, all mentioned points were acknowledged, corrected and precisely reviewed.

**Reviewer: 2**

R2: Thank you for presenting this detailed assessment. I have some comments to help with clarity but overall, excellent presentation.

R2: Please comment on exactly why the Municipal Health Secretary was requested to the specific home? Was this because a resident at the home reconginzed a triatomine? Where they being bitten by teh bug? Chagas disease concerns? Please be more specific and include in "Study area and specimen collection" section.

*A:* Thanks for the relevant issues. The resident of the infested house had already got in touch with the Municipal Health Secretary and also sent us a photo of an insect. By the photo it was possible to identify the insect as a triatomine. Once the insect has been identified we got in touch immediately with the local Municipal Health Secretary together with the Environmental Secretary and we visited residence and proceeded with the searches for triatomines in the residence. Several details were included in the manuscript.

R2: How long was the investigation? Was it for one day? 8 hours? Did they return for multiple entomological assessments? Please put in the details of the investigation timeline.

*A:* We completed the exhaustive searches for triatomines in five hours, including all rooms and the peridomiciliary area. A report was presented to the Municipal Health Secretary together and the Environmental Municipal Secretary. After this, a control spraying was carried out in all compartments of the house. During the treatment and for five days the house was closed, and the residents were asked to moved to another residence. After a week, a second search was carried out to confirm the treatment against the triatomine infestation was effective. Twenty days later a contact with the residents of the infested house confirmed that no more insects were found in the house. All details were included in the M & M including the official technical report sent to the health authorities.

-R2: Figure 2 (D) shows fecal drop on the walls but how was this confirmed? Some insects will defecate on walls and it looks like triatomine feces. I suggest saying, "Inspection of internal structures of the dwelling and observation of suspected triatomine feces on the wall."

*A:* The fecal drops were found in just one of the rooms and it was concentrated in a specific area of the room near the celling. From the out side, the roof was inspected, the tiles were remove on the area were the fecal drops were found. In this inspected area of the roof, nynphs, eggs, exuviae and adults were found. Eventually, triatomines were also found in other rooms of the house as mentioned by the owner. It was evident that in one of the rooms, the triatomines were more frequently found. This point is, now properly addressed in the manuscript.

R2: Did you test for DTU I? Why not?

*A:* At this moment, no DTU was tested. We plan to extend the study in the area and carry out a more detailed search for triatomines and characterization of the *T. cruzi* DTUs; due to the epidemiologic importance of this finding, we decided the turn public the very first results as soon as possible. The perspective of continuation of the scientific research in the area is now presented in the manuscript.

R2: Please provide more details of the blood meal analysis. This is very briefly mentioned. Human blood was detected but documenting the other sources aids in understanding the extent to the other blood meal sources and will also help with the ecology of domestic invasion of a triatomine like this.

*A:* More detailed information was added in the manuscript. We thank the suggestion and we do plan to continue detailed studies in the area.

R2: I like the pictures of the triatomines and where they were found. This is impactful. I found myself wanting to see at least one adult picture which shows clear morphological features of the bug. This would also be impactful for readers.

*A:* We included detailed picture of the *T. vitticeps* that was infesting the house

R2: Please add in the Discussion the known prevalence of Chagas in this locality and in the Rio de Janeiro State.

*A:* Thanks for the suggestions We included additional information on this matter

R2: I would also add some sentences on the impact of a sylvatic vector invading into the peridomestic domain and now domicilatting a home. Why did this occur at this residence? As you mentioned this is a fragmented forest region and deforestation is being seen in many regions of Brasil and other regions where Chagas disease is endemic. Sylvatic and peridomestic vectors are now being seen more and more in these regions. I would make some comments about this and possible site some article displaying this:

*A:* A few lines discussing the domiciliary colonization was added as well as its significance. However, regarding the references below, we'd rather keeping the focus on the *T. vitticeps* findings to avoid a very long discussion session. Also the list of references in the manuscript giving support to the discussion arguments are already encompassing the issues presented in the references below somehow.

R2: Cantillo-Barraza O, Gual-González L, Velásquez-Ortiz N, Medina Camargo MA, González P, Cruz-Saavedra L, Castillo A, Zuluaga S, Herrera G, Cowan H, Velez-Mira A, Patiño LH, Ramírez JD, Triana O, Nolan MS. Triatoma venosa and Panstrongylus geniculatus challenge the certification of interruption of vectorial *Trypanosoma cruzi* transmission by Rhodnius prolixus in eastern Colombia. PLoS Negl Trop Dis. 2025 Jan 27;19(1):e0012822.

R2: Meraz-Medina T, Vences-Blanco MO, Ramírez-Cervantes AG, Nogueda-Torres B, Martínez-Ibarra JA. First record and vital statistics of Triatoma longipennis (Heteroptera, Reduviidae, Triatominae) from Southern Mexico. Acta Trop. 2025 Nov;271:107873.

R2: Beatty NL, Bhosale CR, White ZS, Torhorst CW, Wilson KN, Dorleans R, Stenn TMS, Killets KC, Curtis-Robles R, Burkett-Cadena N, Nováková E, Hamer GL, Hamer SA, Wisely SM. Field evidence of *Trypanosoma cruzi* infection, diverse host use and invasion of human dwellings by the Chagas disease vector in Florida, USA. PLoS Negl Trop Dis. 2025 Jul 7;19(7):e0012920.

R2: Other examples exist. I recommend discussing the importance a bit more as it is a great example of the changing dynamics of Chagas disease within the Americas.

*A:* Thanks. A few more lines on this matter, mentioning the importance of the reported finding was added in the discussion.

Thank you for your work!

*A:* Thanks very much for the nice suggestions; all of them were acknowledged and properly answered.

# Reviewer report: REVIEWERS COMMENTS

## REVIEWER #1

No other comments.

## REVIEWER #2

Authors have provided a much improved manuscript with the recommended additional content from the Reviewers. I have no additional suggestions.
